# AI-mediated cross-cultural translation of Chinese brush-and-ink psychology for aesthetic empathy

**DOI:** 10.3389/fpsyg.2026.1828438

**Published:** 2026-09-08

**Authors:** Bei Tang, Hongying Yan, Yang Li, Yong Hua

**Affiliations:** 1 School of Fine Art and Design, Suzhou University, Suzhou, Anhui, China; 2 School of Psychological and Social Sciences, The University of Waikato, Hamilton, New Zealand; 3 School of Fine Arts, Anhui Normal University, Wuhu, Anhui, China

**Keywords:** aesthetic empathy, AI cross-cultural translation, brush-and-ink psychology, Chinese painting, cross-cultural communication, cultural psychological barrier, psychological symbol

## Abstract

The brush-and-ink system of Chinese painting is a systematic psychological symbol system deeply rooted in Confucian, *Dao*, and *Chan*, where lines, ink tones, and composition, respectively, embody painters’ personality traits, emotional states, and mental patterns. In the context of global digital communication, it faces the dilemma of “visual accessibility but psychological inaccessibility,” mainly due to the obvious East-West cultural-psychological aesthetic gap that seriously hinders Western audiences’ ability to empathize with its implicit psychological connotations. Existing relevant studies mostly focus on the adaptation of visual forms or the simple implementation of AI technology, thus lacking a systematic research framework for the cross-cultural psychological translation of brush-and-ink psychology. Based on cultural psychology and cross-cultural communication theories, this study systematically analyzes the brush-and-ink psychological symbol system, dissects the core differences in East-West aesthetic psychology, and explores AI’s effectiveness in cross-cultural psychological translation across three critical facets: intelligent decoding, visual adaptation, and immersive experience extension. Furthermore, it constructs a three-dimensional pathway model of “brush-and-ink psychology — AI translation — aesthetic empathy,” identifies the core challenges in AI translation practice, and proposes targeted optimization strategies. As an exploratory theoretical research article, this research reveals AI’s core mechanism in bridging cross-cultural aesthetic psychological barriers, fills the existing research gaps, and provides a replicable theoretical and practical model for the international dissemination of Chinese painting’s brush-and-ink psychology and other non-Western art forms.

## 1 Introduction

The psychology of brush and ink revolves around the bidirectional interplay between ink wash painting, a specialized artistic medium, and human psychological processes. Central to this field is the conveyance of Eastern emotional nuances and cultural significances through brushwork. From the creator’s standpoint, this area of study explores how artists’ decisions regarding brush techniques—encompassing variations in stroke pressure and speed, ink tone gradations, and the purposeful employment of negative space—connect to their emotional dispositions, cognitive processes, aesthetic tastes, and engagement with Eastern philosophical thought. In essence, brush and ink give tangible form to the artist’s inner psychological world.

From the audience’s standpoint, this field examines the psychological reactions of viewers—most notably Western audiences—as they perceive and make sense of brushwork. It also explores how the evocative mood of ink wash painting fosters emotional connection and cross-cultural comprehension. This dimension is of great significance, as it lays a psychological groundwork for addressing feedback from Western audiences and promoting cross-cultural communication within the domain of ink wash art.

The ink and brush system of Chinese painting is not merely a technical approach to painting but a culturally embedded psychological symbolic carrier deeply rooted in Confucian, *Dao* (*the Way*), and *Chan* (*meditation*) Buddhist thought. The strength, flexibility, pauses, and turns of lines reflect character and temperament; the density, lightness, dryness, and wetness of ink convey emotional fluctuations; the arrangement of voids and solids in composition reveals psychological structure. This aesthetic characteristic of “ink and brush conveying the mind” constitutes Chinese painting’s unique system of psychological expression. Aesthetic-philosophical concepts such as “rhythmic vitality” (*qiyun shengdong*) and “interplay of void and actuality” (*xushi xiangsheng*) are, in essence, concentrated manifestations of these psychological symbols of ink and brush. These works are tangible expressions of the painter’s inner world, philosophical thinking, and personal experience. They also embody the core of Eastern aesthetic psychology. In this way, they concretize the artist’s spiritual world and life experience, while articulating the profound essence of Eastern aesthetic psychology.

Existing international research has helped advance cross-cultural understandings of Chinese painting esthetics and its ink-brush foundations. These studies include Wen Fong’s (1992) cross-cultural interpretation of “rhythmic vitality,” Zhang and Zhou’s (2025) analysis of visual autonomy in Chinese painting, and Cahill’s (1982) exploration of the cross-cultural construction of ink-and-brush ontology. Later scholarship has further examined the psychological meanings of ink and brush (Kang and Wang, 2025; Wang and Joneurairatana, 2025).

In the globalized digital era, the international dissemination of Chinese painting encounters a new dilemma. While transcending the constraints of physical media, it confronts a critical paradox: visual accessibility yet psychological inaccessibility. For non-Western art forms, this aesthetic barrier of cultural psychology constitutes the core challenge to global communication. The cross-cultural communication of non-Western art commonly encounters empathy deficits at the psychological level. Influenced by their own cultural contexts and aesthetic-psychological models, Western viewers struggle to interpret the implicit emotional expression, metaphorical personality, and conceptual essence behind the ink and brushwork of Chinese painting. The psychological communication dilemma of Chinese ink painting is particularly paradigmatic. Scholars have explored the causes of the lack of cross-cultural empathy for Eastern art, applying theories such as Hall’s (1973/2021) encoding/decoding model, discussing empirical research on barriers to non-Western art dissemination by Ahmed and Poulter (2022), and analyzing reasons for the cross-cultural empathy gap regarding Eastern art, often treating it as a purely visual art form incapable of eliciting deep aesthetic empathy (Guan, 2024; Khan, 2023; Xu X., 2024). Current research focusing on the state of Chinese painting’s international dissemination notes that efforts predominantly remain at the level of visual form, neglecting the translation of the psychological connotations behind the ink and brush (Zhao, 2020). From the perspective of cultural psychology, scholars have conducted in-depth analyses of aesthetic psychological differences between East and West. They have identified a fundamental divide: Eastern esthetics emphasizes implicit comprehension and experiential realization, whereas Western esthetics prioritizes explicit perception (Gao, 2024; Markus and Kitayama, 1991; Wang et al., 2024). This provides theoretical support for the cross-cultural translation of ink-and-brush psychology.

A growing body of research focuses on applying digital technology to the cross-cultural dissemination of non-Western art. In the intersecting field of AI and cross-cultural art communication, international scholarship has begun preliminary exploration, suggesting that technological empowerment can effectively lower barriers to access. However, existing applications predominantly concentrate on the reconstruction of visual forms—such as superficial adaptation, the medium-specific affordances of digital technology, or textual interpretation of aesthetic philosophy—lacking design for psychological-level translation (Šimůnek, 2016; Zabrodskaja, 2025). Some studies focus on AI’s ability for cross-cultural adaptation of artistic forms. Research on AI in art curation by Yan et al. (2025), studies on AI artistic style transfer by Chen and Wang (2025), and explorations in algorithmic extraction of visual aesthetic features by Gatys et al. (2016) validate AI’s effectiveness in the intelligent transformation of visual symbols. Some scholars have attempted to integrate AI with cultural psychology to explore its potential for decoding and translating the psychological substance of art. Bender et al.’s (2021) critique of AI art ethics and translational processes has galvanized a systemic rethinking of technological and social justice. Concurrently, Helliwell (2024) and Li et al. (2020) propose that AI, via big-data mining, offers a pathway to accurately bridging aesthetic perceptions across different cultural landscapes. Other research targets specific practices in AI translation for Chinese painting, focusing on algorithmic simulation and visual representation of ink-brush styles without addressing psychological-level translation. For example, Cheng (2024) explored how digital media technologies can reinterpret and reimagine traditional Chinese landscape painting, while Yang et al. (2025) focused on employing digital tools to preserve and transform the expressive essence of brushwork lines. Furthermore, Zhou et al. (2024) conducted a systematic study on the diversified application of digital technologies in the artistic practices of post-1990s Chinese painters. Furthermore, Cross-cultural communication scholars further contend that fostering “psychological empathy” is imperative for the substantive transmission of non-Western art (Iyer, 2025). Yet, the field currently lacks a robust framework to systematically interpret the psychological dimensions embedded within Chinese ink painting. This has confined cross-cultural communication to “superficial viewing” rather than “deep empathy,” thus hindering the effective transmission of its cultural values.

Digital technology development offers potential solutions to this dilemma. With the ability to mine big data on cultural psychology, intelligently adapt visual symbols, and expand multimodal psychological experiences, AI acts as a key bridge connecting the psychological dimensions of Chinese ink painting with Western aesthetic empathy. Through AI’s algorithmic decoding of personality metaphors in brushstrokes, cross-cultural visual translation of emotional ink tones, and immersive presentation of compositional psychological structures, AI is reshaping the paradigm for cross-cultural dissemination of Chinese painting, shifting its focus from “formal transmission” to “psychological empathy.” Chen (2022) advocates translating Eastern and Western metaphor theories into data-visualization design principles, contending that culturally resonant visual metaphors facilitate the decoding and memorization of complex data among target cultural audiences. Meanwhile, Hung and Ippolito (2020) posits that digital media—including motion pictures and interactive art—offers a novel avenue for reinterpreting and expanding the traditional spatiotemporal logic inherent in Chinese painting and calligraphy. However, a systematic research framework targeting the “translation of ink-and-brush psychology” has not been established, particularly lacking in-depth exploration of AI’s mechanisms and pathways for translation at the aesthetic-psychological level. Existing related studies either emphasize AI’s technical implementation while neglecting the cultural substance of ink-and-brush psychology (Radford et al., 2021), or focus on theoretical discussions of cross-cultural communication while lacking practical pathway design for AI translation (Appadurai, 2013), or concentrate on translating formal aspects of ink and brush while overlooking the guidance of psychological empathy (Wang and Joneurairatana, 2025).

Moreover, international research in the field of psychological translation for non-Western art provides reference and inspiration for this study. Studies on the cross-cultural dissemination of Japanese ukiyo-e or Indian miniature painting propose translation approaches such as “visualization of cultural context” and “adaptation of psychological symbols” (Al Hudib et al., 2016; Bao et al., 2016; Halaszynski, 2025). At the technical level of AI psychological translation, researchers have developed aesthetic preference recognition models based on cultural psychology big data, capable of accurately capturing the aesthetic psychological characteristics of viewers from different cultural backgrounds. Advances in AI-facilitated psychological translation increasingly leverage culture as a key interpretive frame for emotional expression. This has given rise to frameworks capable of modeling the dynamic interplay between macro-cultural norms and individual psychological variation. Concurrently, sophisticated recognition models—trained on cultural big data—are now able to discern distinct aesthetic preferences, accurately reflecting the nuanced responses of viewers from different cultural milieus (Gong et al., 2025; Liang et al., 2026). At the level of cross-cultural empathy assessment, scholars have constructed multi-dimensional empathy evaluation systems, providing methodological support for measuring the effectiveness of ink-and-brush psychological translation. Aparicio (2022) systematically embeds cultural variance into emotion-computing models. Jami et al. (2024) contend that empathy research must operate at two levels simultaneously—the individual (e.g., personal traits, overlapping experiences) and the social (e.g., cultural norms, group standards). Zhang and Noels (2024), in turn, attempt to operationalize the aesthetic philosophy of Chinese painting by breaking it down into measurable cognitive, affective, and behavioral components. However, none of these studies focus specifically on the psychology of Chinese ink painting, making them inapplicable directly to its international dissemination practices and unable to address its core pain point of “lack of psychological empathy.”

This paper, grounded in cultural psychology and cross-cultural communication theory, analyzes the psychological symbolic system of Chinese ink painting, dissects the core differences between Eastern and Western aesthetic psychology, explores AI’s capacity for cross-cultural psychological translation, and ultimately constructs a pathway model of “Chinese Ink Painting Psychology—AI Cross-Cultural Translation—Western Viewer Aesthetic Empathy.” This model aims to provide actionable psychological translation strategies for the international dissemination of Chinese ink painting’s psychological dimensions. It also offers theoretical references and practical insights to help non-Western art overcome cultural-psychological barriers and achieve in-depth cross-cultural communication. It should be noted that this paper represents an exploratory theoretical study. It merely puts forward conceptual models and hypotheses to inform future empirical research, and does not purport to offer definitive empirical conclusions.

## 2 Analyzing the psychological symbolic system of Chinese ink painting: ink conveying mind and psychological expression

Ink and brush in Chinese painting are not merely representational tools, but a comprehensive system of psychological symbols. Its central logic is “ink and brush conveying the mind” (*bimo zaixin*). By carefully controlling lines, ink tones, and composition, the painter externalizes personality, emotions, and psychological structures onto the canvas, establishing the long-standing aesthetic tradition that “painting as the voice of the heart-mind” (*huawei xinsheng*).

The ideal of “fit for wandering and dwelling” (*keyou keju*) is deeply embedded in the painter’s creative psychology. Rooted in Guo Xi’s “Lofty Principles of Forests and Streams” (*lin quan gao zhi*), it embodies the artist’s longing for an ideal life, attachment to nature, and pursuit of spiritual belonging. It stands as both a psychological goal in creation and the ultimate expression of ink-brush psychology.

This integration of aesthetic ideals and creative psychology further strengthens the idea of “inscription of the heart-mind” (*xinyin*) (Fong, 1992). Grounded in traditional Chinese cosmology and life philosophy, this psychological symbolic system is closely linked to Confucian, *Dao*, and *Chan* ideas of self-cultivation. It is the defining feature that separates Chinese painting from Western representational art, and a major reason why Western viewers often find it difficult to achieve genuine aesthetic empathy across cultures.

### 2.1 Line: visual metaphor of personality traits

Chinese painting constructs form through lines. “bone method brushwork” (*gufa yongbi*) is more than a line technique—it visually manifests personality. Line form, strength, and rhythm directly reflect the painter’s character, cultivation, and spirit, laying the basis for Chinese painting’s ink-brush psychological symbols. Rounded, upright, firm, and powerful use of the centered brush tip (*zhongfeng*) metaphorically embodies Confucian “gentleman’s ceaseless self-strengthening” (*junzi ziqiang buxi*) and the “doctrine of the mean” (*zhongyong zhidao*)’s steady demeanor, revealing the painter’s resolve and integrity. Flexible, varied, expansive, and fluent use of the slanted brush tip (cefeng) reflects Daoist “spontaneity and non-action” (ziran Wuwei) (Xu Y., 2024), expressing a transcendent pursuit of following one’s heart (Xiang, 2024). Density, gathering, scattering, pauses, and turns in texturing strokes (*cun, ca, dian, ran*) reveal the painter’s complex personality: spaced lines indicate an inclusive mind; forceful, paused strokes show resilience; smooth, gentle lines convey warmth and humility.

**FIGURE 1:**
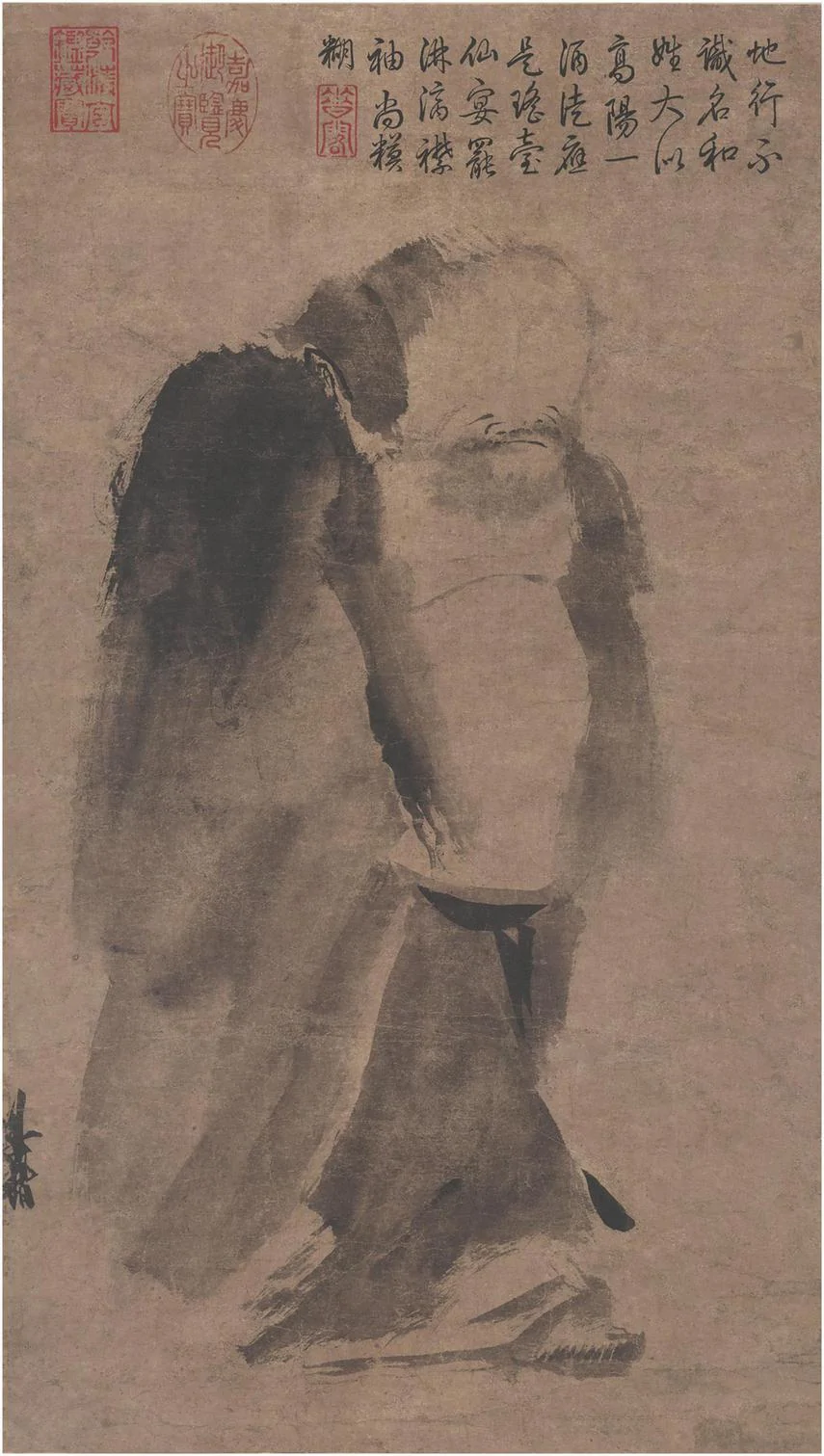
“Immortal in Splashed Ink” was painted by the Southern Song Dynasty painter Liang Kai, and is now in the collection of the Taipei Palace Museum. Image source: https://baike.so.com/doc/554670-587243.html. Reprinted with permission from www.1tu.com (Yitu Network), licensed by Beijing Yitu Houde Network Technology Co., Ltd.

For example, in the Southern Song painter Liang Kai’s “*Immortal in Splashed Ink*” (*Po mo xian ren tu*) (Figure 1), the figure’s outline is sketched with extremely minimal lines. The variation in line thickness and the spontaneous, unforced shifts between solid and void reflect both a Chan Buddhist “seeing one’s true nature” (mingxin jianxing) (Sun, 2025) and metaphorically suggest the painter’s transcendent, unrestrained personality. In Western art, lines prioritize representational accuracy and lack the psychological connotation of “personality metaphor.” As a result, viewers struggle to connect with or empathize with the personality traits conveyed by the lines.

### 2.2 Ink tone: concrete expression of emotional state

The core principle of ink application in Chinese painting is embodied in the “five shades of ink” (*mofen wuse*). The five gradations—charred, heavy, dense, light, and clear—are not merely techniques for depicting texture and spatial depth. Instead, they serve as direct expressions of the painter’s emotional state. Different combinations and applications of ink tones correspond to distinct psychological moods and emotional experiences, establishing a unique system of ink-tone emotional symbolism (Jiang and Li, 2025). Thick, heavy ink often conveys restrained, profound, and solemn sentiments, reflecting the painter’s contemplation and inner feelings. Such ink tones are typically employed in representations of majestic landscapes or dignified figures. Light, ethereal ink corresponds to a peaceful, relaxed, transcendent state of mind, conveying emotions of serenity and detachment from fame and fortune, often used for distant landscapes or expressions of *Chan* emptiness. Dry, parched, and aged charred ink implies resilience, melancholy, and antiquarian emotions, reflecting the painter’s calm and steadfastness after experiencing vicissitudes. Clear, faint, hazy ink conveys tenderness, tranquility, and a sense of distance, creating an ethereal and expansive emotional atmosphere.

**FIGURE 2:**
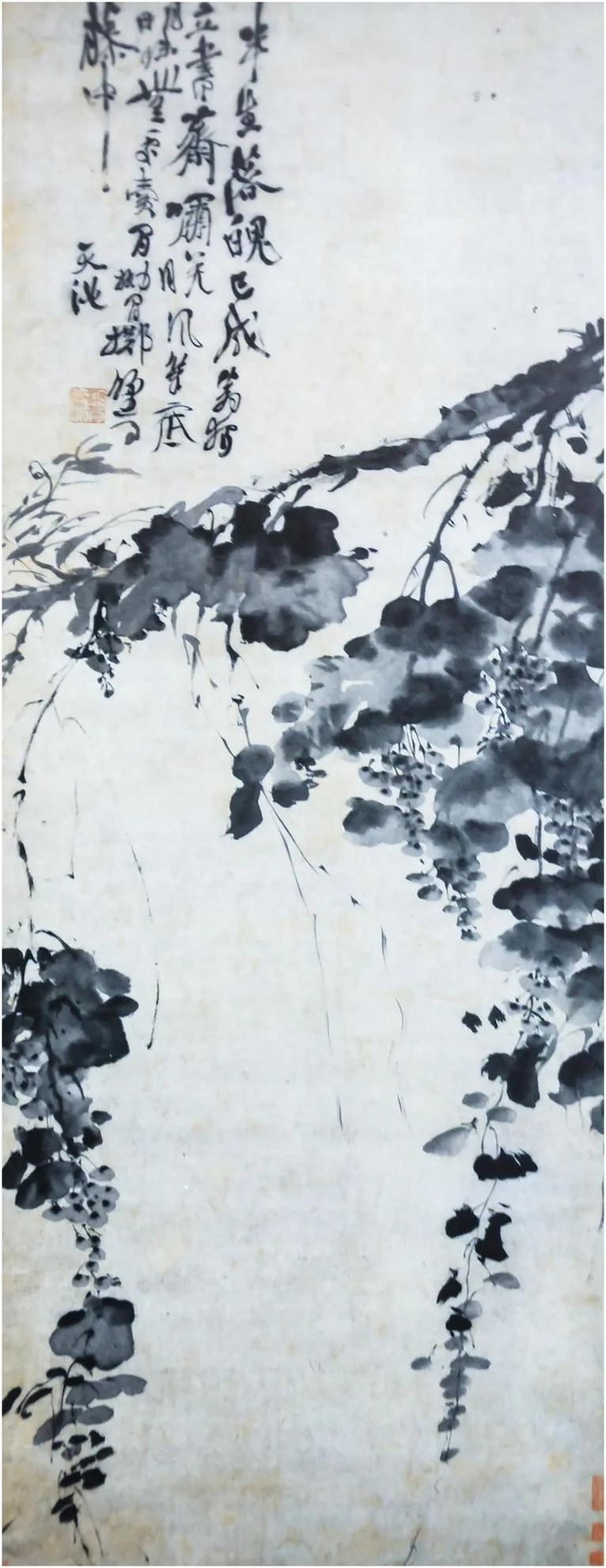
“Scroll Painting of Ink Grapes” is a representative work by the Ming-dynasty painter Xu Wei, and now held in the collection of The Palace Museum in Beijing. Image source: https://digicol.dpm.org.cn/cultural/detail?id=ceddeb0310204940838ca4ad92179f1f&source=6. Reprinted with permission from www.1tu.com (Yitu Network), licensed by Beijing Yitu Houde Network Technology Co., Ltd.

The aesthetic pursuit of “water-ink as superior” (shuimo weishang) (Brubaker and Wang, 2015) in ink painting pushes the emotional expression of ink tone to its extreme. Its subtle and restrained variations in ink tone highly correspond to the Eastern habit of implicit emotional expression—“show neither joy nor anger” (*xi nu bu xing yu se*). In “*Scroll Painting of Ink Grapes*” (*Moputao Zhou*) (Figure 2), Xu Wei of the Ming dynasty eschews overt emotionalism — allowing the forms of the grapes and vines themselves to suggest the inner sentiment. The painter’s emotional state is articulated through the subtle nuances of ink shades. This mode of implicit emotional expression stands in stark contrast to the direct emotional representation typical of Western art. Accustomed to interpreting emotion through pronounced color contrasts and exaggerated formal expressions, Western viewers often struggle to discern the subtle affective connotations latent in Chinese ink tones. This creates an empathy deficit at the affective level.

### 2.3 Composition: spatial presentation of psychological structure

Composition in Chinese painting is not merely the arrangement of objects, but a spatial expression of the painter’s psychological structure and cosmology. Principles such as “treating white areas like black ink” (*jibai danghei*), “interplay of void and actuality,” and the “three distances” (*san yuan*) are essentially spatial externalizations of the painter’s inner world, conveying the unique psychological cognition and value pursuits of Eastern people. The “leaving blank space” (*liubai*) composition of “treating white areas like black ink” considers the blank (void) and depicted forms (solid) equally important psychological elements. The blank is not emptiness but an extension of the painter’s psychological space—it can be an emotional pause or an expansion of mental state, reflecting the painter’s dialectical psychology of “mutual generation” and an open, inclusive psychological structure. The compositional approach of the “three distances” integrates the spatial layers of “high distance,” (gaoyuan) “deep distance,” (shenyuan) and “level distance,” (pingyuan) breaking the limitations of physical space, conveying the painter’s reverence for nature and the cosmos, and the psychological pursuit of “harmony between humanity and nature” (tianren heyi), embodying a grand psychological structure of “embracing the world” (Yang, 2020). The interplay of density, concentration, and dispersion mirrors the painter’s innate sense of order and equilibrium. A composition marked by rhythmic variation and harmonious balance reveals a mind that is calm, restrained, and composed.

**FIGURE 3:**
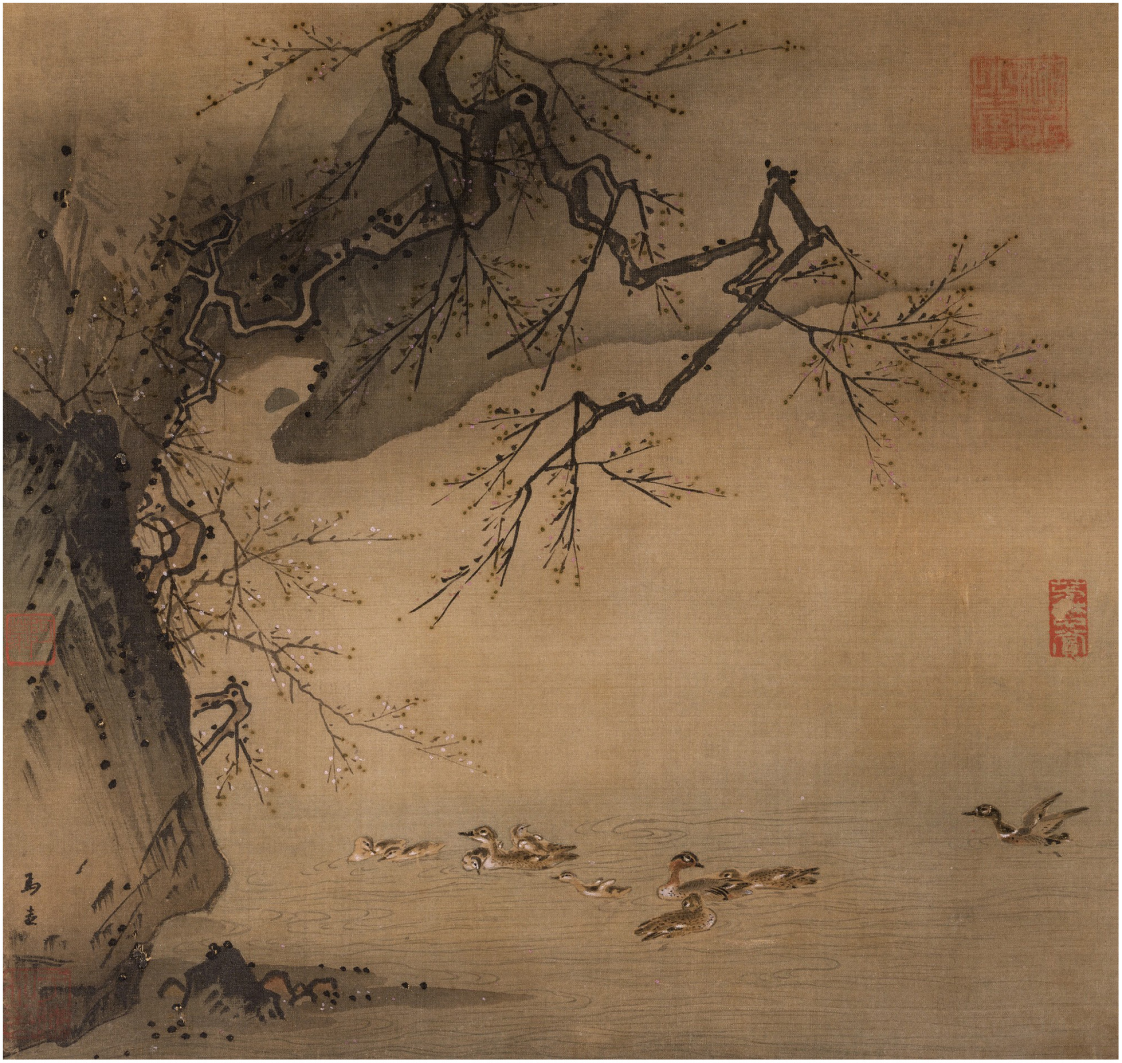
“Wild Ducks in a Mountain Stream” is a painting by the Southern Song Dynasty painter Ma Yuan, and is now in the collection of The Palace Museum in Beijing. Image source: https://www.dpm.org.cn/collection/paint/234350.html. Reprinted with permission from www.1tu.com (Yitu Network), licensed by Beijing Yitu Houde Network Technology Co., Ltd.

Unlike the realistic, single-point perspective composition of Western painting, Chinese composition emphasizes the creation of “psychological space” over the reproduction of physical space. Its core lies in conveying the painter’s psychological feelings and spiritual pursuits through spatial arrangement. This characteristic aligns closely with the creative psychology of “fit for wandering and dwelling” (Gu, 2014). For example, it can be seen from the Southern Song Dynasty painter Ma Yuan’s “*Wild Ducks in a Mountain Stream*” (*Mei shi xi fu tu*) (Figure 3) that the painter has always been psychologically oriented to “fit for wandering and dwelling” in his creations, constructing pictorial space imbued with both natural charm and spiritual warmth through ink-brush composition. “Wandering” manifests as a state of mental freedom during creation; the painter “roams” within the picture using ink and brush as media, transforming personal experiences of travel and feelings of freedom into the expansiveness of lines and the liveliness of ink. “Inhabiting” reflects the desire for a sense of belonging during creation; the painter constructs a habitable spiritual homeland through ink and brush, integrating inner peace and aspirations for an ideal life into the arrangement of void and solid and the warm texture of ink tones (Zhang and Zhou, 2025). This deep integration of “psychological composition” and the “wanderable and habitable” creative psychology requires the viewer to interpret it through an Eastern mode of psychological cognition. Influenced by a “realist” aesthetic psychology, Western viewers often focus on the physical plausibility of composition, overlooking its underlying psychological substance and the original creative intent of “wanderable and habitable,” thus struggling to understand the psychological structure and spiritual pursuit conveyed by the composition.

## 3 East-West differences in aesthetic psychology: the core barrier to cross-cultural empathy

Culture and psyche are not discrete; they are mutually constitutive. Culture frames cognition, while psychology drives cultural evolution. Therefore, understanding the mind requires situating it within its historical milieu, just as analyzing a culture demands interrogating the psychological dynamics of its members.

Given the heterogeneity of mental processes across backgrounds, cross-cultural communication must transcend linguistic translation. It requires engaging with the substratum of values, cognitive patterns, and social norms. By rejecting the absolutism of stereotypes and respecting both universal patterns and individual agency, we can achieve a nuanced understanding of the “other” and genuinely transcend cultural boundaries.

The core obstacle in the international dissemination of Chinese ink painting’s psychology is not the difference in visual form but the fundamental divergence between Eastern and Western aesthetic psychology (Gu, 2021). Eastern aesthetic psychology centers on “implicitness, experiential realization, and dialectics,” emphasizing psychological experience and spiritual resonance during the aesthetic process. Western aesthetic psychology centers on “explicitness, concreteness, and rationality,” stressing visual perception and logical cognition. This divergence in aesthetic psychology leads Western viewers to struggle in forming corresponding psychological cognition and emotional resonance when interpreting the psychological symbols of Chinese ink painting, creating the “aesthetic barrier of cultural psychology” in cross-cultural communication (Kitayama and Salvador, 2024), which is also the core issue unresolved by existing cross-cultural dissemination research.

### 3.1 Eastern aesthetic psychology: implicit comprehension, emphasizing “Psychological Resonance”

Rooted in Confucian, *Dao*, and *Chan* traditions, Eastern aesthetic psychology centers on implicit comprehension. It views aesthetic experience not simply as visual observation, but as internal realization and spiritual resonance. It follows the ideal of “obtaining the meaning and forgetting the image” (*deyi wangxiang*): the viewer penetrates artistic forms to grasp their psychological essence and spiritual value, and thus reaches the state of “fusion of object and self.” In viewing Chinese painting, Eastern audiences do not stop at the surface of brush and ink. They bring their own cultural mentality and life experience to interpret the personality in lines, the emotions in ink tones, and the psychological structure in composition. Through such contemplative realization, they attain psychological resonance with the painter (Kang and Wang, 2025). This aesthetic trait is deeply tied to the Eastern worldview of “harmony between humanity and nature,” and forms a key feature that sets it apart from Western aesthetic psychology.

This aesthetic psychology arises from Eastern cultures’ emphasis on implicit, restrained emotional expression and dialectical, holistic thinking. In Eastern traditions, people tend to eschew direct self-expression, instead conveying inner states through indirect, suggestive means. The psychological dimension embedded in Chinese ink painting serves as a distinct artistic manifestation of this cultural disposition. In tandem, the cosmology of “harmony between humanity and nature” encourages an orientation toward the intrinsic connections and dialectical unity of all phenomena (Tang and Li, 2024). Under this worldview, the formal qualities of brushwork and ink are regarded as dynamically intertwined and mutually reflective of the artist’s state of mind. The aesthetic experience thus centers on apprehending these internal relationships and attaining genuine psychological resonance (Leung, 2021). Such a psychology-centered, non-explicit aesthetic framework lends Chinese ink painting a high degree of implicitness and subjectivity. In turn, it also elevates the challenges inherent in its cross-cultural translation — a view supported by existing empirical research in cross-cultural esthetics.

### 3.2 Western aesthetic psychology: explicit, concrete, and centered on visual perception

Rooted in the realist tradition of ancient Greece and rationalist philosophy, Western aesthetic psychology is characterized by explicitness and concreteness. It places central emphasis on visual perception and logical cognition throughout aesthetic experience, prioritizing precision and tangibility in artistic form while pursuing formal verisimilitude as a key aesthetic goal. Within this framework, the aesthetic subject acquires clear perceptual information through direct engagement with formal features, enabling the formation of rationally grounded aesthetic judgments (Funch, 2021). When engaging with art, Western viewers typically focus on the accuracy of visual components such as form, color, light, and shadow, and favor the direct expression of emotion and conceptual meaning (Gombrich, 2023). Consequently, they often find implicit, ambiguous, or indirect modes of psychological expression difficult to interpret (Liu, 2022). This orientation aligns with the rationalist philosophical tradition that stresses external observation and logical analysis.

The formation of such aesthetic psychology is closely intertwined with Western cultural tendencies toward direct, outward emotional expression and rational-analytical modes of thought. Western cultures generally prioritize straightforward articulation of personal feelings and self-expression, reflected in artistic practices that often employ strong color contrasts, pronounced formal exaggeration, and explicit narrative structures to convey emotions and ideas directly. Correlatively, rationalist thought orients viewers toward the external forms and formal logic of objects (Afifah, 2023; Felgner, 2023), assuming that artistic value derives primarily from the faithful rendering of the physical world. This makes it challenging for them to grasp the subtle, implicit psychological logic underpinning concepts such as the interplay of void and substance or “grasping the spirit beyond the image” in Chinese ink painting. They may thus struggle to perceive the personality, emotional layers, and structural psychology embedded within brushwork and ink tones (Mutiani et al., 2022). Such fundamental cognitive disparities constitute a core source of the empathy deficit observed in East-West aesthetic encounters.

### 3.3 Specific manifestations of the aesthetic psychology barrier: core causes of empathy deficit

Cross-cultural dissemination of Chinese ink painting is constrained by a persistent cultural-psychological barrier, arising from deep-rooted divergences in Eastern and Western aesthetic psychology that translate into a multi-layered empathy deficit. At the level of personality metaphor, Western audiences tend to perceive the linear forms in Chinese ink painting purely as representational tools, rather than as symbolic expressions of the artist’s character and temperament. This prevents them from recognizing the personality metaphors inscribed in brushwork and from achieving genuine identification with the painter. In the domain of emotional expression, viewers raised within Western aesthetic conventions are accustomed to explicit and direct affective representation. They often fail to discern the subtle emotional nuances embedded in the delicate gradations of ink tone, leaving them unable to grasp the painter’s inner affective world or attain emotional empathy. Regarding cognitive and structural comprehension, Western aesthetic perception is strongly anchored in focal-point perspective and mimetic realism, which leaves little room for the “paradox-embracing” philosophical thinking that is central to other traditions (Deguchi et al., 2021). This makes it challenging to appreciate the unique psychological space and structural logic embodied in compositional principles such as “valuing blankness as substance” (Wang and Wang, 2025) and the “three distances” (Wu, 2025). As a result, viewers struggle to engage with the artist’s spiritual pursuits and cosmological outlook, obstructing the formation of profound spiritual empathy.

Existing cross-cultural art dissemination research mostly focuses on superficial adaptation of visual forms, attempting to adapt to Western viewers’ visual aesthetic habits by adjusting the visual appearance of ink and brush or simplifying composition (Jiang et al., 2021). However, this “symptomatic not curative” approach fails to address the core at the aesthetic-psychological level, cannot break the cultural-psychological barrier, and struggles to achieve Western viewers’ deep empathy for Chinese ink painting psychology, ultimately leading cross-cultural communication into the predicament of “superficial viewing” (Morphy, 2007). This further confirms that the cross-cultural dissemination of Chinese ink painting psychology must be based on East-West aesthetic psychology differences and construct targeted psychological translation strategies.

The foregoing analysis points to real and consequential differences, yet it would be a mistake to treat these East-West aesthetic orientations as rigid binaries. Historical scholarship has often cast the contrast as one between Western mimesis—a rationalist mode rooted in optical and geometric principles—and Eastern *qi yun*, that elusive quality of spirit resonance and inner vitality. But dichotomies of this kind tend to obscure more than they clarify. A more productive view is to see these orientations as fluid spectra, defined by emphasis rather than opposition. In contemporary practice, this binary is increasingly giving way to hybrid models. AI, in this context, does more than shuttle between two fixed positions. It opens up a new space of aesthetic experience, where the geometric precision of Western perspective meets the atmospheric fluidity of Eastern composition. The outcome is not a synthesis that absorbs both, nor a middle ground between them, but an emergent aesthetic register irreducible to either lineage.

## 4 The efficacy of AI in cross-cultural psychological translation: core support for breaking aesthetic empathy barriers

In the realm of cross-cultural art communication, the core advantage of AI technology lies in its ability to mine, analyze, and intelligently adapt to big data. Its intelligent interpretation ability based on cultural psychology big data, cross-cultural adaptation to visual forms, and extension of multimodal psychological experience can accurately connect the psychological symbols of Chinese ink painting with the aesthetic psychology of Western audiences, realizing the cross-cultural transformation of brush-and-ink psychology. This makes artificial intelligence a core support for breaking down aesthetic psychological barriers and promoting aesthetic empathy among Western audiences.

### 4.1 Intelligent interpretation: precise decoding of ink-brush psychological symbols

The collection and preprocessing of data call for rigorous standards and robust scientific protocols. To secure a diverse and representative corpus, the dataset ought to encompass Chinese ink paintings across historical periods and stylistic lineages, supplemented by relevant archival materials. Biographical information on the painters should also be systematically organized and integrated into the training phase as contextual cues, which in turn provides a solid foundation for decoding accuracy.

Decoding the psychological dimensions of ink wash painting requires the systematic extraction of both brushwork and compositional features. For brushwork analysis, convolutional neural networks can be deployed to quantify line thickness, curvature, density, and ink properties, including tonal variation, dryness, and wetness, and to generate high-dimensional feature representations. Image saliency detection can then be used to locate key brushstroke regions; deep learning would further extract their texture, tone, and spatial characteristics. Affective computing techniques could then link these features to established emotional categories, such as joy, sadness, or calm, offering a viable path toward the systematic interpretation of emotional expression.

On the compositional front, a multi-tiered approach may be adopted: image segmentation first divides the painting into distinct regions, from which geometric attributes, including area, position, and shape, are derived. Graph-theoretic models would then be used to analyze the spatial relationships among these regions. To ground these visual features in psychological constructs, they can be correlated with pre-annotated categories, such as personality traits or emotional states, derived from expert labeling, with art historians and psychologists working in tandem, and informed by established taxonomies in affective science as well as traditional Chinese aesthetic theory, including the “Six Principles” (*liufa lun*) and the “*Xieyi.*” Through iterative refinement of the mapping rules, the correspondence between formal brushwork features and psychological interpretations can be progressively sharpened, providing a robust theoretical foundation for future empirical validation.

Recent work has used AI to study traditional Chinese calligraphy, uncovering systematic links between brushwork and the writer’s psychological profile—trait emotional intelligence, for instance, can be read from stroke dynamics and angular transitions (Lyu et al., 2025). Other studies have taken a longitudinal approach, finding a “lonely and transcendent” emotional register in lighter-ink works and a measurable relationship between ink density and emotional intensity (Zhou et al., 2024). Yang et al. (2025) go a step further, connecting such brushwork to East Asian cultural norms of emotional restraint and offering Western viewers a way into the symbolic language of ink. What emerges from these studies is a clearer path through a longstanding challenge in cross-cultural esthetics: how to make the psychological dimensions of brushwork legible across cultural lines.

### 4.2 Visual adaptation: cross-cultural translation of ink-brush psychology

From the perspective of psychological symbolism in Eastern and Western cultures, brush and ink serve as a medium for Chinese literati to express inner cultivation and moral integrity—embodied in the notion that “the calligraphy reflects the person”—and to connect with the natural cosmos through “Rhythmic Vitality.” The gradations of ink, from light to dark and wet to dry, alongside variations in line quality—ranging from swift to slow, light to heavy—are all projections of the artist’s state of mind and vitality. In contrast, Western audiences may perceive brush and ink merely as an exotic “artistic tool” with a unique visual aesthetic. They tend to focus more on line texture, abstract forms, and compositional effects, rather than the profound cultural and ethical connotations beneath them.

A comparison of the Western pursuit of representation, perspective, and chiaroscuro with the Eastern reverence for freehand expression, negative space, and artistic conception reveals that these differences stem from distinct cognitive modes: Western analytical thinking versus Eastern holistic thinking. As a quintessential high-context cultural product, Oriental ink wash art relies heavily on the viewer’s prior understanding of Chinese philosophy (Daoism, *Chan*), literature (poetry), and history (the literati painting tradition). Consequently, for audiences from low-context cultures lacking this background, decoding its profound meaning poses a significant challenge. Based on the precise decoding of ink-brush psychological symbols, AI intelligently adapts the visual forms of Chinese ink painting to the aesthetic psychology of Western viewers. It converts implicit ink-brush symbols into explicit, concrete visual expressions that Western audiences can easily understand and accept. This strategy achieves the cross-cultural translation goal of “retaining psychological substance while adapting visual form” and overcomes empathy barriers in visual perception. Visual adaptation functions in two key ways. It translates personality metaphors embedded in brush lines into explicit visual cues such as textual annotations and color coding. Warm or cool tones further highlight traits like gentleness or resolve, helping Western viewers establish direct links between line characteristics and personality. As for emotional expression in ink tones, AI strengthens subtle emotions through color overlay and light-shadow modulation. This makes the emotional content embedded in ink layers more intuitive and accessible across cultures. For instance, adding a faint gray overlay to thick, heavy ink to enhance a sense of oppression, or adding soft lighting effects to light, ethereal ink to enhance tranquility, allowing Western viewers to directly capture emotions in ink through visual perception. AI also clarifies the psychological significance of blank spaces in compositions guided by the principle of “treating white areas like black ink.” With gentle light-shadow outlines or subtle visual hints, it reveals the inherent psychological logic of such compositions. It further translates the spatial conception of the “three distances” into three-dimensional spatial representations familiar to Western viewers, allowing them to comprehend the deeper psychological structure embedded in the composition.

For example, Cheng (2024) proposes an AI-driven model for translating traditional Chinese painting across cultures. It reworks the classical “scattered perspective” into immersive formats—narrative-driven experiences like 3D animation, or freely explorable ones like VR. The model operates on two fronts: esthetically, it uses AI style transfer to bridge Eastern and Western visual idioms; conceptually, it transcribes urban data into landscape forms. The model retains the expressive energy of the original brushwork while offering legible interpretive cues—“spontaneous brushwork,” for instance, is read as “spiritual autonomy.” Spiritual autonomy, as understood here, refers to a creative state in which the artist’s brushwork and formal decisions arise from inner conviction rather than from external conventions or established rules. For Western audiences, the term may evoke the Romantic celebration of individual expression and artistic liberty. Yet it does not imply unbridled self-indulgence. On the contrary, it designates a discipline-based spontaneity—one shaped by years of practice and grounded in Confucian and Daoist ideals of self-cultivation and the mutual belonging of humanity and nature.

This clarification allows Western viewers to approach the work’s psychological dimensions more directly, perceiving line-based personality cues and compositional intentions through visual means. Such perceptual access, in turn, opens the way toward emotional and spiritual resonance. The resulting mode of visual adaptation preserves the psychological core of Chinese ink painting while making its visual language more legible to audiences familiar with explicit aesthetic expression—thus helping to close the empathy gap at the level of perception.

### 4.3 Experience extension: multi-modal pathways to deepened psychological empathy

AI technology integrates AR/VR, interactive design, and other multi-modal tools to extend the cross-cultural experience of ink-brush psychology. Through this integration, immersive scenarios for psychological empathy can be effectively established, deepening Western viewers’ perception and resonance and enabling a progressive transition from visual perception to psychological experience and ultimately to emotional resonance. When combined with VR technology, AI creates immersive environments for Chinese painting appreciation. Western viewers are able to “step into” the painting and intuitively grasp the essence of ink-brush psychology through interactive operations—such as perceiving structural strength by touching brush lines or interpreting emotional shifts by adjusting ink tones—thereby enhancing their overall psychological engagement. On the other hand, AI combined with interactive design can adjust the visual form of ink and brush in real-time based on viewers’ emotional feedback. For instance, adjusting ink density based on the viewer’s heart rate changes, enabling emotional interaction with the artwork and strengthening emotional resonance. Simultaneously, AI can provide multilingual voice interpretation of psychological content, helping Western viewers understand the cultural context of ink-brush psychological expression, deepening psychological empathy.

With the deep integration of art and science, more and more scientists are paying attention to the transformation and application of art and neuroaesthetics research results. In June 2025, Professor Gao Xiaorong’s team at the Tsinghua University Neuroengineering Laboratory advanced the frontiers of neuroaesthetics by conducting a series of experiments in the ecologically valid setting of the New Tsinghua Auditorium. Utilizing portable, non-invasive electroencephalography (EEG) for simultaneous multi-subject acquisition, the study interrogated the neural underpinnings of aesthetic appreciation elicited by the classical dance drama Only This Green—a masterpiece embodying the literati ethos of traditional landscape painting (Tsinghua University, 2025).

The research decoded the neural correlates of emotional resonance, revealing that immersive live performances trigger robust, whole-brain synchronization closely coupled with affective distribution. Notably, significant physiological modulations were observed even among lay audiences lacking professional artistic training. Beyond characterizing the neural dynamics of art appreciation, this work establishes a feedback loop of “evaluation-re-creation,” thereby providing a rigorous empirical framework for optimizing artistic creation, refining arts education, and developing novel interventions for emotional disorders.

## 5 Core challenges facing AI cross-cultural psychological translation

Despite AI technology offering effective solutions for cross-cultural translation of Chinese ink painting psychology, three core challenges remain in practice, constraining translation effectiveness and the formation of aesthetic empathy. First, insufficient depth in ink-brush psychological decoding. Current AI decoding of ink-brush psychology mostly “form-psychology” surface correspondence, struggling to capture the deep cultural-psychological substance and individual psychological differences of painters behind the ink and brush, leading to imprecise and less rich psychological expression after translation. Second, difficulty in the “degree” of cross-cultural adaptation. Visual perception varies across different cultural contexts, and the influence of cultural values, religious beliefs, historical traditions, and social norms on psychological experience is equally diverse. Conversely, these variations in psychological experience also give rise to differences in emotional expression. Over-adaptation to Western aesthetic psychology may weaken the core characteristics and psychological substance of Chinese ink painting, leading to homogenization or Westernization. Western audiences continue to face strong aesthetic and psychological barriers under inadequate adaptation, which severely restricts the emergence of genuine cross-cultural empathy. The absence of a structured empirical framework for psychological empathy also makes it difficult to quantify viewers’ affective engagement with Chinese ink painting, leaving translation strategies without a reliable basis for further improvement.

## 6 Future development pathways

### 6.1 Constructing a pathway model for AI cross-cultural translation of Chinese ink painting psychology

**FIGURE 4:**
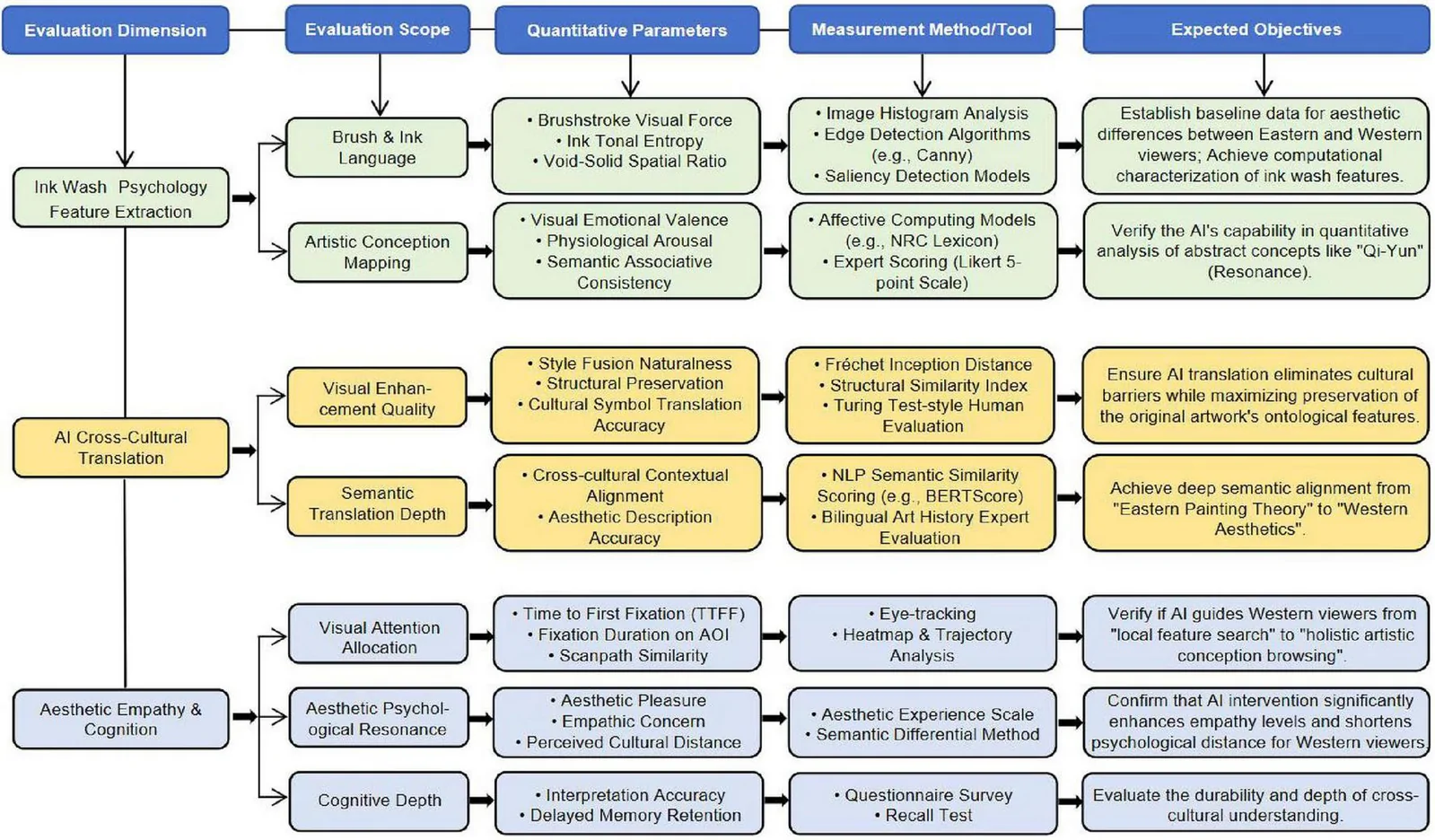
Evaluation index system of AI cross-cultural translation path model.

Given AI’s demonstrated strengths and persistent limitations, this study proposes a three-dimensional pathway model: Chinese Ink Painting Psychology—AI Cross-Cultural Translation—Eastern/Western Viewer Aesthetic Empathy. The model is rooted in the psychological symbolism of Chinese ink painting, East-West aesthetic divergences, and AI’s capacity for cross-cultural psychological translation. With psychological empathy as its core, the model employs AI as the translational medium, centers on ink-wash psychological symbols, and uses East-West aesthetic differences to guide adaptation. In this model, aesthetic empathy is embodied as a correlation index between visual attention allocation, aesthetic psychological resonance and cognitive depth. Psychological symbols are embodied in the evaluation weight of brush and Ink language on the psychological mapping of artistic conception. This method enables abstract artistic psychological phenomena to be mapped and calculated by AI algorithms in high dimensions, thus realizing the transformation from perceptual experience to quantitative model (as shown in Figure 4).

This study extract explicit morphological features—texture, stroke strength, and rhythmic dynamics—from artistic brushstrokes. We take these extracted morphological features as the input space, with dimensional scores from standardized psychological scales as the output space. Leveraging the intrinsic logic of cross-space mapping, the framework constructs a directional, hierarchical, and quantifiable association mechanism—one that effectively converts low-level visual features (i.e., brush-and-ink morphology) into high-level psychological traits. Through quantitative calibration, we optimize the mapping path, thereby further enhancing the stability and accuracy of this cross-space association. Ultimately, this approach—rooted in mapping logic—yields a high-precision affective decoding model. AI enables precise decoding of ink-wash esthetics, capturing the character of brushlines, the emotion of ink tones, and the psychological structure of composition. Visual forms are adapted to Western aesthetic psychology, translating implicit psychological symbols into explicit, concrete expressions. Multi-modal technologies deepen psychological engagement and build immersive empathy contexts. For Western viewers, visual perception evolves into psychological experience, and emotional resonance deepens into spiritual identification. This sequence nurtures profound aesthetic empathy and enables the successful cross-cultural transmission of psychological meanings in Chinese ink painting.

The model presents three distinct strengths. It prioritizes psychological translation to overcome the “form over psychology” bias inherent in traditional cross-cultural communication. As an AI-driven framework, it enables precise, intelligent, and personalized translation of ink-wash psychology. Customized solutions can be designed to match the aesthetic profiles of diverse Western viewers. For example, style transfer algorithms and image segmentation techniques are used to process line fluency and ink tonal gradations (density, lightness, wetness, and dryness), respectively. To enhance the visual impact of brush lines for Western audiences, contrast enhancement algorithms are applied. Regarding differentiated strategies, the system provides high-fidelity restoration of the original for researchers, while offering accessible visual guidance to general viewers through augmented reality (AR) overlay annotations, color mapping, and other approaches. The model also integrates a complete operational chain: decoding, adaptation, experience, and empathy. This structure ensures effective translation and advances cross-cultural communication from superficial viewing to deep, enduring empathy.

The model’s value manifests at two levels. Practically, it provides actionable psychological translation frameworks for the global dissemination of Chinese ink painting. These frameworks effectively transcend cultural-psychological barriers, foster deep empathetic engagement among Western audiences, and enhance the international communication capacity and influence of Chinese painting. Theoretically, it fills the research gap concerning “psychological translation strategies” in existing cross-cultural art communication studies, constructs a research paradigm of “ink-brush psychology—AI translation—aesthetic empathy,” and offers theoretical references and practical insights for non-Western art to surmount cultural-psychological barriers and achieve in-depth cross-cultural communication.

### 6.2 Optimization directions

#### 6.2.1 Deepening ink-brush psychological decoding, mining deep cultural-psychological substance

Future research should advance AI’s capacity to decode ink-wash psychology and its surface correspondences, with emphasis on excavating the profound cultural-psychological essences and individual psychological differences inherent in painters’ brushwork. A foundational step involves expanding ink-wash psychology big data infrastructure through the establishment of a “Chinese Ink-Wash Painting Psychological Database,” which integrates extensive Chinese paintings, artists’ biographies, and cultural-psychological research.

Specifically, this database should cover works across dynasties, artistic styles, and painters, alongside aesthetic psychological data from diverse cultural contexts.

Linking ink-wash artistic forms to their corresponding psychological impacts can be significantly enhanced by incorporating artists’ life experiences, cultural backgrounds, and psychological states, which in turn improves the accuracy of AI-based decoding and adaptive interpretation. Furthermore, implementing personalized fine-tuning mechanisms within data training frameworks and integrating limited samples from individual painters allows for effective accommodation of stylistic heterogeneity across artists.

Furthermore, optimizing cross-cultural translation algorithms is critical to enhancing the dynamic detection of Eastern-Western aesthetic discrepancies. Leveraging deep learning and natural language processing, we can develop an intelligent model linking ink-wash forms to their psychological essence. This model enables the accurate identification and extraction of brushstroke features, ink-tone emotions, and compositional psychology, ensuring translated expressions remain faithful, layered, and consistent with the core spirit of Chinese ink-wash painting.

Multimodal technologies offer a critical pathway to extending cross-cultural experiences of ink-wash psychology. Emerging technologies—including the metaverse and brain–computer interfaces—can construct immersive, empathy-rich environments. By deepening perceptual engagement among Western audiences, they enhance cross-cultural communication and help mitigate distortions in cultural semiotics. For instance, VR immersive scenarios can enable Western viewers to enter pictorial spaces and engage with their psychological connotations via interactive operations.

To complement these multimodal and technical efforts, an AI-driven interactive feedback system can dynamically adjust ink-wash expressions and interpretations in real time based on viewers’ emotional and behavioral responses, supporting personalized psychological experiences. Integrating multilingual psychological interpretation via concise cultural-psychological explanations further clarifies the contextual meanings of ink-wash expressions and deepens understanding.

#### 6.2.2 Balancing the “Degree” of cross-cultural adaptation, achieving harmony between tradition and modernity

“Imagery” (yixiang), a core aesthetic of Chinese painting, can be computationally rendered into “illusions” (huanxiang) (Xu and Liu, 2022) that resonate with Western audiences via AI mediation. Instead of leaving cross-cultural viewers to guess at the artist’s emotional state, AI can step in with accessible explanations, bringing in cultural context or case studies to reveal the painter’s working conditions and mindset. This enables audiences across cultures to connect not only with the painter’s emotions but also with the philosophical and spiritual layers embedded in the work.

Accordingly, cross-cultural visual adaptation calls for preserving psychological substance while adapting form. Careful calibration of adaptation ensures coherence between the intrinsic qualities of Chinese ink painting and Western aesthetic psychology. Core features of Chinese ink painting are retained, including the vitality of lines, the layering of ink tones, and the poetic conception of composition. Excessive adaptation is avoided to prevent the erosion of authentic ink-wash expression. The “adaptable elements” of perspective logic in composition, color saturation, and linear structure of narrative are visually adapted to Western aesthetic preferences for explicitness, concreteness, and direct perceptual experience. Implicit psychological meanings are translated into accessible and acceptable visual forms, enabling Western audiences to appreciate the unique charm of Chinese ink painting with “visual readability.” In this way, an organic integration of traditional essence and modern expression is achieved.

Textual prompts, color assistance, and line enhancement may be used to help Western viewers associate brushlines with character traits. Color overlays, light and shadow variations, and emotional annotations strengthen the emotional expression of ink tones for immediate comprehension. Compositional ideas such as “leaving blank space” and the “three distances” are translated into three-dimensional spatial structures and intuitive psychological cues familiar to Western viewers. This supports deeper understanding of the psychological substance behind compositional design.

#### 6.2.3 Constructing a psychological empathy evaluation system, optimizing translation strategies

A rigorous and systematic evaluation framework for psychological empathy is established to quantify Western viewers’ empathetic responses to Chinese ink painting psychology, supporting evidence-based optimization of translation strategies. For professional audiences, the AI implements a “weak adaptation” strategy, prioritizing preservation of the original work’s psychological depth in brush and ink with only semantic-level auxiliary explanations (e.g., AR annotations). In contrast, for general audiences, the system adopts a “strong adaptation” strategy focused on visual resonance. This is accomplished by modulating color contrast and superimposing concrete visual guidance layers to lower the cognitive threshold. The framework comprises three interrelated dimensions: visual perception, measuring audience acceptance of translated visual expressions; psychological experience, assessing comprehension and experiential depth of ink-brush essence; and emotional resonance, evaluating the extent of emotional and spiritual identification. Empirical data are collected via questionnaires, behavioral analysis, and affective monitoring. Quantifiable metrics—eye gaze duration and facial expression change frequency—were employed to assess the visual perception dimension. Depth of understanding was evaluated through psychological tests and interviews, with the validity of the criteria confirmed using quantifiable empathy measures, such as the degree of emotional mimicry and similarity in emotional experiences.

Given the inherent characteristics of the three-dimensional psychological empathy assessment system, this study could adopt an integrated methodological framework, incorporating questionnaire analysis, behavioral experimentation, and physiological monitoring. Empirical data are systematically collected through standardized questionnaires, objective behavioral analysis, and quantitative affective monitoring, thereby ensuring the entire assessment procedure is well-organized and rigorously standardized. AI-enabled analytics identify translational limitations and support iterative improvements. A complementary psychological empathy guidance system is also proposed. AI-driven recommendation engines deliver personalized content aligned with Western aesthetic preferences, facilitating gradual understanding of Eastern implicit expression. Methods and outcomes of cultural sensitivity training for promoting awareness of cross-cultural differences in visual perception, psychological experience, and emotional resonance, and for enhancing intercultural communication competence.

This research outlines methods for analyzing assessment outputs to drive targeted refinements in AI translation algorithms, specifically by optimizing affective lexical weighting and integrating cultural frameworks. Furthermore, we propose strategies to enhance adaptive mechanisms, focusing on the dynamic calibration of translation styles to accommodate diverse cultural contexts. Cross-cultural platforms encourage interactions between Western audiences and Chinese artists, fostering deeper empathy and advancing the integration of Eastern and Western aesthetic psychology.

#### 6.2.4 Fostering interdisciplinary and international collaborative innovation

AI-enabled cross-cultural psychological translation of Chinese ink painting psychology represents an interdisciplinary and international systematic endeavor. Success relies on integrated efforts across art studies, cultural psychology, computer science, cross-cultural communication, and related disciplines. Dedicated research platforms may be established to strengthen interdisciplinary collaboration. Key issues including ink-brush psychological decoding and cross-cultural adaptation can be addressed through joint efforts by artists, psychologists, computer engineers, and communication scholars. Multicultural education exerts a positive significance in fostering cross-cultural understanding and inclusiveness, and in promoting the exchange and integration of diverse cultures. International cooperation and academic exchange may be further advanced. Joint projects with Western universities, research institutions, and art organizations allow translation strategies to be refined in accordance with Western aesthetic psychology. Mutual understanding and integration of Eastern and Western aesthetic systems are thereby promoted, establishing a favorable environment for the global dissemination of Chinese ink painting psychology.

Core AI translation technologies, including deep learning and multimodal fusion, demand high-standard equipment and technical personnel. For small and medium-sized art communication institutions, resource integration in art communication requires pragmatic solutions. Renting on-demand computing resources from cloud service providers cuts equipment acquisition and maintenance costs effectively. Collaboration with peers or relevant entities, meanwhile, facilitates shared technical resources and expertise to collectively address technical challenges. To mitigate talent shortages, institutions may conduct internal training or partner with universities and research institutions for targeted technical personnel cultivation.

## 7 Conclusion

Our inquiry focuses on the “aesthetic barrier of cultural psychology”—a pivotal constraint in the cross-cultural communication of non-Western art—through which we delineate the unique psychological symbolism of Chinese ink painting, identify fundamental disparities in Eastern and Western aesthetic cognition, and evaluate AI’s capacity to facilitate cross-cultural psychological translation. Drawing on these analytical insights, we propose an integrated pathway model: “Chinese Ink Painting Psychology—AI Cross-Cultural Translation—Western Viewer Aesthetic Empathy.” Our findings provide innovative psychology-centered strategies for the global communication of Chinese ink painting, alongside robust theoretical references and practical insights for non-Western art traditions seeking to overcome cultural-psychological barriers and cultivate profound, authentic cross-cultural dialog. Cultural integration in cross-cultural communication is not ink wash art’s one-sided concession to Western esthetics. Instead, leveraging Western audience feedback, it achieves “Eastern genes + Western expression” alignment, preserving the art’s native essence while ensuring Western understanding and acceptance. Western feedback on “contemporaneity,” “innovation,” and “interactivity” drives ink wash art beyond traditional models, innovating in media, form, and ideology to infuse contemporary vitality and forge a globally communicable contemporary ink wash language.

Our results demonstrate that ink and brush in Chinese painting operate as a comprehensive psychological symbolic system, rather than merely serving as technical implements: lines correspond to personality, ink tones to emotional states, and composition to psychological structure—all anchored in the aesthetic ideal of “ink expressing the mind.” Human “perceptual esthetics” is the core of ink wash art, and AI “rational decoding” serves as its auxiliary tool. In practice, balancing these two elements relies on the two-way pathway of “technology empowering perception”—this mutual reinforcement not only meets Western audiences’ demand for contemporaneity and innovation but also upholds the native essence of ink wash art, aligning with its global communication needs. AI enables intelligent decoding, visual adaptation, and multimodal experience extension—a capability that effectively bridges ink-brush psychology and Western aesthetic perception, facilitates the cross-cultural translation of Chinese painting, and transforms communication from formal transmission to genuine psychological empathy.

This study is subject to several inherent limitations. To begin with, the proposed framework remains theoretical rather than empirical. Consequently, the mechanisms governing AI-driven cross-cultural psychological translation are hypothetical rather than proven. In the absence of data, we cannot confirm the causal claims posited by the model. Furthermore, the psychological empathy evaluation system remains in a preliminary stage. In light of these gaps, future research must prioritize the empirical testing of this framework. Specifically, controlled studies are required to gather data, verify mechanisms, and establish a robust evaluation system. Only through such validation can these theoretical pathways be substantiated.

## Data Availability

The original contributions presented in this study are included in this article/supplementary material, further inquiries can be directed to the corresponding author.
